# Management of Breast Abscess during Breastfeeding

**DOI:** 10.3390/ijerph19095762

**Published:** 2022-05-09

**Authors:** Paola Pileri, Alessandra Sartani, Martina Ilaria Mazzocco, Sofia Giani, Sara Rimoldi, Gaia Pietropaolo, Anna Pertusati, Adriana Vella, Luca Bazzi, Irene Cetin

**Affiliations:** 1 Department of Woman, Child and Neonate, Buzzi Children Hospital, ASST Fatebenefratelli Sacco, Via L. Castelvetro 32, 20154 Milan, Italy; martina.mazzocco@libero.it (M.I.M.); irene.cetin@unimi.it (I.C.); 2 Departmental Breast Unit, ASST Fatebenefratelli Sacco, University of Milan, Via G.B. Grassi 74, 20157 Milan, Italy; sofigiani@gmail.com; 3 Department of Biomedical and Clinical Sciences, University of Milan, Via G.B. Grassi 74, 20157 Milan, Italy; alessandra.sartani@asst-fbf-sacco.it (A.S.); gaia.pietropaolo@asst-fbf-sacco.it (G.P.); annapertusati@gmail.com (A.P.); 4 Laboratory of Clinical Microbiology, Virology and Diagnostics of Bioemergencies, ASST Fatebenefratelli Sacco, University of Milan, Via G.B. Grassi 74, 20157 Milan, Italy; sara.rimoldi@asst-fbf-sacco.it; 5 Department of Radiology, “Luigi Sacco” University Hospital, Via G.B. Grassi 74, 20157 Milan, Italy; adriana.vella@asst-fbf-sacco.it (A.V.); luca.bazzi@asst-fbf-sacco.it (L.B.)

**Keywords:** breast abscess, breastfeeding, needle aspiration, surgery

## Abstract

(1) Background: Breast abscess (BA) is a condition leading in the majority of cases to breastfeeding interruption. Abscesses are commonly treated with antibiotics, needle aspiration or incision and drainage (I&D), but there is still no consensus on the optimal treatment. Since there are no well-defined clinical guidelines for abscess management, we conducted a retrospective, observational study with the aim of assessing ultrasound (US)-guided management of BA without surgery, regardless of the BA size. The secondary objective was the microbiologic characterization and, in particular, the *S. aureus* methicillin resistance identification. (2) Methods: our population included 64 breastfeeding mothers with diagnosis of BA. For every patient, data about maternal, perinatal and breastfeeding features were collected. All patients underwent office US scans and 40 out of 64 required a more detailed breast diagnostic ultrasound performed by a radiologist. In all cases, samples of milk or abscess material were microbiologically tested. All patients received oral antibiotic treatment. We performed needle aspiration, when feasible, even on abscesses greater than 5 cm. (3) Results: most of the women developed BA during the first 100 days (68.8% during the first 60 days) after delivery and 13 needed hospitalization. Four abscesses were bilateral and 16 had a US major diameter greater than 5 cm. All patients were treated with antibiotic therapy according to our clinical protocol and 71.9% (46/64) underwent fine needle aspiration. None of them required I&D. The average duration of breastfeeding was 5 months (IR 2; 9.5) and 40.6% of women with BA continued to breastfeed for more than 6 months. Only 21 mothers interrupted breastfeeding before 3 months. (4) Conclusions: our observational data suggest, regardless of the size and the clinical features of the BA, a conservative approach with antibiotic therapy targeted at the Methicillin-Resistant *Staphilococcus aureus* (MRSA) identified and needle aspiration, if feasible. In our experience, treatment with needle aspiration is a cost- effective method. Unlike drainage, it is an outpatient procedure, easily repeatable, with no cosmetic damage. In addition, it has lower risk of recurrences since, differently from surgical incision, it does not cause interruption of the ducts. Moreover, needle aspiration is less painful, does not require the separation of the mother-child dyad and allows for a quicker, if not immediate, return to breastfeeding.

## 1. Introduction

Breastfeeding is the earliest form of communication between mother and child and breast milk is the best food for infants, species-specific, recommended by major societies, such as WHO [1], UNICEF [2], American College of Obstetricians and Gynecologists (ACOG) [3] and American Academy of Pediatrics (AAP) [4]. It has positive effects on mothers and their breastfed babies, enduring throughout life [5]. It provides all the nutrients needed in the first phase of life and contains bioactive and immunological substances that are not found in artificial substitutes. It promotes mother–child bonding, contributing to the increase in the intellectual quotient (IQ) and, through oxytocin production, stimulates the natural uterine contractions, reducing post-partum bleeding [4,5,6,7,8]. These are the reasons why the World Health Organization (WHO) recommends exclusive breastfeeding for the first 6 months of the infant’s life, and continued breastfeeding up to 2 years and beyond [1]. In a study performed by our group in 2014–2016, we found that breastfeeding support and promotion are the most significant factors that could affect breastfeeding outcomes [9]. 

During breastfeeding, problems or diseases may arise that may compromise its success if not promptly and appropriately treated. If during breastfeeding the mother reports pain, the presence of breastfeeding breast disease should be suspected and the most frequent causes are mastitis and breast abscesses. Breast abscess (BA) is a serious condition, related to severe morbidity in lactating women leading in the majority of cases to breastfeeding interruption with all its consequences. BA are defined as localised areas of infection with a walled-off collection of pus [10]. It may or may not be associated with mastitis, which represents its most severe complication. BA develops in 3% to 11% of women with mastitis with reported incidence of 0.1% to 3% in breastfeeding women. Cases due to *Staphylococcus aureus* are the most common and the majority of isolated strains are resistant to penicillins [11]. A progressive increase of breast infections due to Methicillin-resistant *Staphylococcus aureus* (MRSA) is reported, but few data are available regarding its real incidence. It varies among different countries in the world (e.g., <5% in UK, 60% in United States) [12,13]. In 2019, Rimoldi et al. published a study conducted in Italy where MRSA strains were responsible for 50% of breast abscesses in lactating women [14]. Less common are cases due to coagulase-negative staphylococci and streptococci [11]. Risk factors for BA are: advanced maternal age at delivery, primiparity, gestational age greater than 41 weeks, previous mastitis, cracked nipples, breastfeeding difficulties during hospital stays and working mothers [15]. The diagnosis of breast abscess is clinical and is confirmed by ultrasound [16]. Ultrasonography is the baseline radiologic technique to diagnose a BA, which results in a hypoechoic or anechoic mass surrounded by a hyperechoic area due to edema [17,18]. Abscesses are commonly treated with antibiotics, ultrasound-guided needle aspiration or incision and drainage (I&D), but there is still no consensus on the optimal treatment. When I&D is performed, the abscess is cut open with a scalpel to release the infected fluid, while treatment by needle aspiration is less invasive. Using ultrasound (US) guidance, a needle (18–19 Gauge) is inserted into the cavity of the breast abscess and a syringe is used to draw out the infected fluid [19]. Several authors have reported surgical incision with drainage as the first-line therapy for abscesses with a size greater than 3–5 cm or multilocular [20,21,22]. However, surgery necessitates local or general anesthesia, separation of the mother from her baby, and is a major risk of ending breastfeeding and scarring with its cosmetic outcomes. Moreover, scars in the breast tissue represent a major risk for further BA. A proper I&D must be performed in the operating room and requires hospitalization, with consequent higher costs [23,24]. Many recent studies support the treatment of lactational breast abscesses with needle aspiration, with or without US guidance [23,25,26]. A timely diagnosis and adequate treatment are essential, as mastitis and abscess represent one of the main reasons that lead to early weaning, with the loss of the benefits that derive from this practice for mother and child. Furthermore, if inadequately treated, they can lead to the development of sepsis and occasionally be fatal [27,28,29].

For a better management of these pathologies we created, in our II level medical center in Milan (ASST Fatebenefratelli—Sacco), a multidisciplinary team, composed of gynaecologists, breast surgeons, radiologists, pediatricians, microbiologists, midwives and nurses. Given that abscess management is still controversial, we conducted this retrospective observational study with the aim of assessing US-guided management of BA without surgery, regardless of BA size. A secondary objective was the microbiologic characterization and, in particular, the *S. aureus* methicillin resistance identification.

## 2. Materials and Methods

This retrospective, observational study was performed at the Azienda Socio Sanitaria Territoriale (ASST) Fatebenefratelli—Sacco in Milan, between January 2016 and December 2019. Our population included 64 breastfeeding mothers with breast abscesses. The diagnosis of lactational breast abscess was made in the presence of clinical inflammatory signs (pain, redness, inflammatory skin) and often a localized, pulpable breast lump was present. The clinical picture was associated with an ultrasound finding of a localised area of infection with a walled-off collection of pus. All our patients underwent office ultrasound for the diagnosis of BA performed by a gynecologist or a breast surgeon. Forty out of the 64 patients required a more detailed breast diagnostic ultrasound performed by a radiologist to better characterize the lesion and understand if it was a drainable abscess cavity. All patients were followed by the multidisciplinary team according to the therapeutic diagnostic protocol in force. Every woman signed an informed consent for invasive procedures. For every patient recruited, data about maternal, perinatal and breastfeeding features were collected. We obtained information on breastfeeding outcomes by means of telephone interviews carried out after six months from the childbirth. Follow up was not possible for 5 mothers. The BA size was measured by ultrasound when performed by the radiologist. We established 5 cm as a cut off to distinguish between small and large abscesses. This parameter was chosen since many authors claimed that abscesses larger than 5 cm should be treated with I&D [11,16,22]. In all cases, samples of milk or abscess material, or both, were sent for microbiological testing in the laboratory. According to the Academy of Breastfeeding Medicine’s (ABM) clinical protocol, milk samples were collected by manual pressing of the breast following the cleansing of the skin, the nipple, the areola, and the operators’ hands [10]. An intermediate milk sample was collected for a total of 5–10 cc. The abscess purulent material was collected by needle aspiration or surgical drainage of the affected area. Needle aspiration was carried out after adequate disinfection of the skin, preparation of a sterile field, and local anesthesia with Lidocaine spray, using an 18 Gauge needle and a 20 cc syringe. I&D is a procedure that involves the injection of anesthetic into the intradermal tissues with a 25- or 30-Gauge needle followed by an incision directly over the center of the abscess. The goal is to allow sufficient space to introduce hemostats, to break up loculations and to place internal packing material. The wounds drain spontaneously but sometimes require gentle pressing to empty the residual content. Samples were collected in a sterile urine culture container, transferred by a sterile syringe to the BacT/ALERT blood culture bottle for Anaerobics (BioMérieux, Marcy L’Etoile, France) and sent to the Laboratory of Clinical Microbiology, Virology and Bioemergecy of the ASST. The samples were analysed with the automated BacT/ALERT microbial detection system, and the positive ones were grown in selective agar plates. The identification of the microbial species was carried out by mass spectrometry with MALDI-TOF technology (BioMérieux, Marcy L’Etoile, France), and the antibiogram was performed with the Vitek 2.0 automatic analyser (BioMérieux, Marcy L’Etoile, France), according to the EUCAST (European Committee on Antimicrobial Susceptibility Testing) breakpoints. Since a high prevalence of MRSA in BA was found, as demonstrated by a previous study from the same group, all patients infected with this bacterium received oral antibiotic treatment (Clindamycin 300 mg 4 times a day for 10–14 days), based on the antibiogram [14]. When feasible, abscesses greater than 5 cm were also treated by needle aspiration using the same procedure described for performing the culture examination on the purulent material. To evaluate the feasibility of performing such a procedure, we considered the ultrasound characteristics of the abscess (mainly liquid content) and the clinical examination (perception on palpation). Whenever possible, such a procedure was preferred because of the immediate benefit to the patient and the potential of a faster resolution. However, the dimensions of 3 cm were maintained as a cut off for surgical treatment, as described in literature [20,21,22] whenever the characteristics described above were not considered as fully met. All the analyses were performed using the statistical software SPSS. The qualitative characteristics were described using the absolute frequencies in each category. The quantitative characteristics were described using mean and standard deviation (SD) or median and the interquartile range. The significance of the differences between the study groups was calculated with a Student’s *t*-test for continuous variables and with the χ^2^ test for categorical variables. Two-tailed *p*-values < 0.05 were considered statistically significant. The success of the needle aspiration treatment was estimated by the proportion of abscesses that recovered without resorting to surgical drainage and by the proportion of patients who did not stop breastfeeding. 

## 3. Results

The socio-demographic and obstetrical data of the population included in this study are listed in Table 1. Primiparity and vaginal birth were an important feature in this population. Exclusive breastfeeding at diagnosis was present in 51.6% while 54.7% of women used breastfeeding aids at diagnosis. Most of the women developed BA during the first 100 days (68.8% in the first 60 days) after delivery and 13 needed hospitalization. Thirty-four women had fissures, 4 had bilateral abscesses and 16 BA had a US major diameter greater than 5 cm (Table 2). Women with BA < 5 cm and >5 cm were similar in the characteristics analysed (Table 3). In addition, no significant differences in socio-demographic characteristics of patients between the analysed groups were observed (data not shown). All the BA > 5 cm were *S. aureus* positive; among these, 56% were methicillin-resistant. All patients were treated with antibiotic therapy according to our clinical protocol and 71.9% (46/64) underwent fine needle aspiration. None of them required I&D. The average duration of breastfeeding was 5 months (IR 2; 9.5) and 40.6% of the women continued to breastfeed for more than 6 months (Table 4). The most common microorganism identified was *Staphylococcus aureus* (*n* = 58): 55.2% were Methicillin-sensitive *Staphylococcus aureus* (MSSA) (*n* = 32) and 44.8% were MRSA (*n* = 26). Among women who had a cesarean section, the proportion of patients with MRSA infection was significantly greater than MSSA (38.5% vs. 9.4%, *p* < 0.05), confirmation that recent surgery is a risk factor for MRSA infection, as stated in the WHO “*MRSA surviving network*” [30]. There were no other differences between the two groups, regarding socio-demographic, clinical and obstetrical features (Table 5 and Table 6). 

## 4. Discussion

This is the first Italian observational study about the management of breast abscesses during breastfeeding by a multidisciplinary team. According to previous studies, the primiparity and recent surgery appear to be associated with the development of breast abscesses [15]. The difficulty to start breastfeeding after surgery and the prevalence of microorganisms with antibiotic resistance during the hospitalization were the probable factors of BA occurring during the puerperal period [30]. The difficulty in breastfeeding also resulted in the use of breastfeeding aids by 54.6% of the lactating mothers. 

Antibiotics and I&D were considered as standard management of breast abscesses up until the early 1990s, after which US-guided interventions became the preferred approach, but there is still no consensus in literature regarding the optimal management of large and multilocular BA. A prospective study, published in *Breast*, regarding 45 women with lactational BA who were randomly treated with either needle aspiration or I&D, showed that all I&D patients were treated successfully, but 70% of them were not satisfied with the cosmetic outcome. On the other side, in the needle aspiration group, 41% of women did not heal following the procedure and an abscess size larger than 5 cm was identified as a risk factor for failure of the procedure [16]. In another prospective study conducted in 30 women with breast abscesses treated by needle aspiration of pus, oral antibiotics, and repeated aspiration (if necessary), 18 patients required only a single aspiration, 9 patients required multiple aspirations, and 6 patients required incision and drainage (overall cure rate, 82%). The patients in whom needle aspiration was successful had a significantly smaller volume of pus on initial aspiration (4.0 mL versus 21.5 mL, *p* = 0.002) [31]. However, consistent data and randomized trials in the literature are limited and a Cochrane review published in 2014 stated that there was insufficient evidence to determine whether needle aspiration is a more effective option than I&D for lactational breast abscesses [19]. In addition, *BMJ Best Practice* published in 2017 suggests that incision and drainage should be reserved for patients in whom aspiration failed and/or for large abscesses (>5 cm in diameter) [11]. However, some recent studies have suggested that the treatment of BA with needle aspiration should be preferred to surgery, regardless of the BA size. A Cameroonian study, published in 2020, enrolled 28 patients diagnosed with lactational breast abscesses, treating them with aspiration and oral antibiotics, and eventually with instillation of ceftriaxone. The study showed that 76% of the patients continued breastfeeding after abscess treatment [25]. In our population, 64.3% of patients continued breastfeeding for more than 3 months. Moreover, Colin et al. published a study reporting that US-guided percutaneous management was successful in 96% of the cases (101/105), regardless of BA size, and allowed continued breastfeeding [23]. Results from a recent retrospective pilot study, published in 2021, including 28 patients with diagnosis of lactational BA and managed by US guided aspiration as first line therapy, showed that a single aspiration was sufficient in 64.3% of the cases, that there were no differences in size of abscesses between patients receiving needle aspiration alone and those who have undergone surgery (*p* = 0.97), that patients who had been managed by needle aspiration continued breastfeeding after the treatment and 40% of the patients were still breastfeeding at 6 months [32]. Moreover, in the largest single study published, which evaluated 151 breast abscesses (lactational and non- lactational) treated with US-guided drainage, 86 (97%) out of 89 patients with puerperal abscesses recovered after the first round of ultrasound-guided drainage [33]. In our study, all patients were treated with safe oral antibiotics during breastfeeding and 71.9% underwent fine needle aspiration. None of them required I&D. The average duration of breastfeeding was 5 months (IR 2; 9.5) and 40.6% of women with BA continued to breastfeed for more than 6 months (64.3% for more than 3 months). We confirm that the main pathogen was *S. aureus* (90%) with a methicillin-resistance in 56% of BA > 5 cm. Recurrences occurred in nine patients treated with antibiotics and needle-aspiration. There were 6 primiparous and 4 of them needed hospitalization. In addition, 50% of them were due to MRSA, the other 50% to MSSA. These BA developed in women previously treated for mastitis with an inadequate antibiotic therapy or in women in whom breastfeeding was inadequately suspended. This suggests that there could be a correlation between these factors and BA, but this needs to be further investigated. In the clinical practice, we do not recommend interrupting breastfeeding during the acute phase in order not to worsen the clinical condition and not to favour relapses. Following our results, regardless of size and clinical features of BA, we suggest a conservative and multidisciplinary approach with antibiotic therapy based on the MRSA prevalence and needle aspiration, if necessary. Our study reports the data collected from a relatively significant number of cases, and the execution of microbiological tests based on bacterial cultures allowed us to perform targeted antibiotic therapy in all our patients. This kind of diagnostic-therapeutic approach promotes healing and above all allows mothers to continue breastfeeding, as demonstrated by the fact that more than 40% of the women in our study continued to breastfeed for more than 6 months.

On the other hand, an important limit of the study is that we have not standardized the clinical and ultrasound parameters for evaluating a breast abscess. This is in part due to the fact that often they are “emergency-urgency” situations in which ultrasound tests were carried out by the medical personnel attending the patient and not by radiologists. The US were performed in the emergency department with an office ultrasound equipment in order to rapidly assess the need to be drained. Other limitations of the study are the fact that it is a retrospective study and that we lost some patients in the follow up. In addition, a limitation of the study is that we could not analyse the correlation between the time point at which BA occurred with socio-demographic and obstetrical data (no data available). This could be an interesting aim for a future study because exploring this relationship could be helpful for prediction. However, our retrospective study has suggested that needle aspiration may be performed, regardless of the BA size and characteristics, in more patients than previously thought and avoid the surgical procedure of I&D. It also allowed us to check accurately the microorganisms in the abscess material aspirated and target the antibiotic therapy. Such preliminary observations would require a confirmation by a controlled study.

## 5. Conclusions

In our experience, treatment with needle aspiration of BA in breastfeeding women is a cost-effective method for many reasons. Unlike incision and drainage, it is an outpatient procedure, easily repeatable, with no cosmetic damage and potentially lower risk of recurrences. In addition, it is cheaper because it does not require the use of operating rooms and hospitalization. Moreover, needle aspiration is less painful, does not require the separation of the mother–child dyad and allows a quicker, if not immediate, restart of breastfeeding. In addition, it is advisable to treat breast abscesses in referral centers, where patients are managed by a multidisciplinary team. 

## Figures and Tables

**Table 1:** Socio-demographic and obstetrical characteristics.

	Population (*n* = 64)
**Age (years)**	33.07 ± 6.99
**Smoking (%) (*n*)**	1.6 (1)
**BMI (kg/m^2^)**	22.2 ± 3.9
**Marital status** - Married (%) (*n*) - Unmarried (%) (*n*) - Unknown (%) (*n*)	50 (32) 26.6 (17) 23.4 (15)
**Educational qualification:** - Degree (%) (*n*) - High school diploma (%) (*n*) - Secondary school diploma (%) (*n*) - Unknown	46.9 (30) 23.4 (15) 7.8 (5) 21.9 (14)
**Parity** - Primiparous (%) (*n*) - Multiparous (%) (*n*)	81.2 (52) 18.8 (12)
**Gestational age at birth (weeks)**	39.3 ± 1.4
**Pregnancy onset** - Spontanous (%) (*n*) - ART (Assisted Reproductive Technology) (%) (*n*)	93.7 (60) 6.3 (4)
**Mode of delivery** - Cesarean section (%) (*n*) - Vaginal birth (%) (*n*)	23.4 (15) 76.6 (49)
**Birthweight (g)**	3241.5 ± 421.5
**Sex of newborn** - F (%) (*n*) - M (%) (*n*)	51.6 (33) 48.4 (31)
**Breastfeeding at birth** - Exclusive (%) (*n*) - Complementary (%) (*n*) - Unknown (%) (*n*)	53.1 (34) 21.9 (14) 25 (16)
**Breastfeeding at diagnosis** - Exclusive (%) (*n*) - Complementary (%) (*n*) - Unknown (%) (*n*)	51.6 (33) 40.6 (26) 7.8 (5)
**Use of breastfeeding aids at diagnosis (breast pump, nipple shilds)**	54.7 (35)

Data expressed as mean ± SD and %.

**Table 2:** Clinical characteristics.

	Population (*n* = 64)
**Days between birth and diagnosis (*n*)**	35 [25.25; 58.75]
**BA developed in the first 60 days (%) (*n*)**	68.8 (44)
**Fissures (%) (*n*)**	53.1 (34)
**Concurrent diseases (candidiasis, vasospasm) (%) (*n*)**	6.3 (4)
**Hospitalization (%) (*n*)**	20.3 (13)
**Bilateral abscesses (%) (*n*)**	6.3 (4)
**Abscesses > 5 cm (%) (*n*)**	25 (16)

Data expressed as median and IQR and %.

**Table 3:** Clinical and microbiological characteristics of patients compared by the size of abscess.

	Abscess < 5 cm (*n* = 24)	Abscess > 5 cm (*n* = 16)
**Days between delivery and diagnosis (*n*)**	34.5 [25; 58.25]	35 [25.25; 58.75]
**Fissures (%) (*n*)**	58.3 (14)	43.7 (7)
**Concurrent patologies** **(vasospasm, candidiasis) (%) (*n*)**	4.5 (1)	6.2 (1)
**Hospitalization (%) (*n*)**	8.3 (2)	43.7 (7)
**Bilateral abscesses (%) (*n*)**	8.3 (2)	12.5 (2)
**Fine needle aspiration (%) (*n*)**	62.5 (15)	87.5 (14)
**Culture examination—Bacteria** - *S. aureus* (%) (*n*) - Not *S. aureus* (%) (*n*)	79.2 (19) 20.8 (5)	100 * (16) 0 (0)
**Antimicrobial resistances** - MRSA (%) (*n*) - MSSA (%) (*n*) - Others (%) (*n*) - No resistences (%) (*n*)	37.5 (9) 37.5 (9) 12.5 (3) 12.5 (3)	56.2 (9) 37.5 (6) 0 (0) 6.3 (1)

Note: data expressed as median and IQR and %. Significance: Student’s *t*-test and ꭓ^2^ analysis; * *p* < 0.05.

**Table 4:** Follow up—breastfeeding duration.

	Population (*n* = 64)
Lost at follow up	5
	***n* = 59**
**Breastfeeding duration** - <3 months (%) (*n*) - 3–6 months (%) (*n*) - >6 months (%) (*n*) - >12 months (%) (*n*)	35.7 (21) 23.7 (14) 20.3 (12) 20.3 (12)
**Breastfeeding duration (months)**	5 [2; 9.5]
**Weaning (months)**	5.355 ± 2.09
**Recurrences (%) (*n*)**	15.2 (9)

Data expressed as median and IQR, mean ± SD and %.

**Table 5:** Socio-demographic and obstetric features of patients with *S. aureus* infection.

	MSSA (*n* = 32)	MRSA (*n* = 26)
**Age (years)**	32.3 ± 5.1	33.5 ± 9.0
**Smoking (%) (*n*)**	0 (0)	3.8 (1)
**BMI (kg/m^2^)**	22.5 ± 4.1	22.3 ± 4.5
**Mode of delivery** - Vaginal birth (%) (*n*) - Cesarean section (%) (*n*)	90.6 (29) 9.4 (3)	61.5 (16) 38.5 * (10)
**Exclusive breastfeeding at birth (%) (*n*)**	71.9 (23)	42.3 (11)
**Breastfeeding at diagnosis** - Exclusive (%) (*n*) - Complementary (%) (*n*) - No breastfeeding (%) (*n*)	65.6 (21) 31.2 (10) 3.2 (1)	46.1 (12) 38.5 (10) 15.4 (4)
**Use of breastfeeding aids (breast pump, nipple shilds) (%) (*n*)**	50 (16)	57.7 (15)

Data expressed as mean ± SD and %. Significance: Student’s *t*-test and ꭓ^2^ analysis; * *p* < 0.05.

**Table 6:** Clinical features of patients with *S. aureus* infection.

	MSSA (*n* = 32)	MRSA (*n* = 26)
**Days between birth and diagnosis (*n*)**	34 [25; 54]	34.5 [25.25; 56]
**Fissures (%) (*n*)**	59.4 (19)	42.3 (11)
**Concurrent diseases** **(vasospasm, candidiasis) (%) (*n*)**	3.1 (1)	3.8 (1)
**Hospitalization (%) (*n*)**	21.9 (7)	23.1 (6)
**Bilateral abscesses (%) (*n*)**	6.2 (2)	7.7 (2)
**Abscesses > 5 cm (%) (*n*)**	21.9 (7)	34.6 (9)

Data expressed as median and IQR and %. Significance: Student’s *t*-test and ꭓ^2^ analysis.

## Data Availability

The data presented in this study are available on request from the corresponding author. The data are not publicly available due to privacy.
